# Efficacy and safety of hydrokinesitherapy in patients with dystrophinopathy

**DOI:** 10.3389/fneur.2023.1230770

**Published:** 2023-07-26

**Authors:** V. M. Suslov, L. N. Lieberman, P. G. Carlier, G. N. Ponomarenko, D. O. Ivanov, D. I. Rudenko, G. A. Suslova, E. I. Adulas

**Affiliations:** ^1^Department of Rehabilitation, Federal State Budgetary Educational Institution of Higher Education Saint-Petersburg State Pediatric Medical University of the Ministry of Healthcare of the Russian Federation, Saint Petersburg, Russia; ^2^University Paris-Saclay, CEA, Frédéric Joliot Institute for Life Sciences, SHFJ, Orsay, France; ^3^Federal State Budgetary Institution Federal Scientific Center of the Rehabilitation of the Disabled Named After G. A.Albrecht of the Ministry of Labour and Social Protection of the Russian Federation, Saint Petersburg, Russia

**Keywords:** Duchenne muscular dystrophy, rehabilitation, physical therapy, hydrokinesis therapy, muscle MRI

## Abstract

Duchenne muscular dystrophy (DMD) is one of the most common forms of hereditary muscular dystrophies in childhood and is characterized by steady progression and early disability. It is known that physical therapy can slow down the rate of progression of the disease. According to global recommendations, pool exercises, along with stretching, are preferable for children with DMD, as these types of activities have a balanced effect on skeletal muscles and allow simultaneous breathing exercises. The present study aimed to evaluate the effectiveness of regular pool exercises in patients with Duchenne muscular dystrophy who are capable of independent movement during 4 months of training. 28 patients with genetically confirmed Duchenne muscular dystrophy, who were aged 6.9 ± 0.2 years, were examined. A 6-min distance walking test and timed tests, namely, rising from the floor, 10-meter running, and stair climbing and descending, muscle strength of the upper and lower extremities were assessed on the baseline and during dynamic observation at 2 and 4 months. Hydrorehabilitation course lasted 4 months and was divided into two stages: preparatory and training (depend on individual functional heart reserve (IFHR)). Set of exercises included pool dynamic aerobic exercises. Quantitative muscle MRI of the pelvic girdle and thigh was performed six times: before training (further BT) and after training (further AT) during all course. According to the results of the study, a statistically significant improvement was identified in a 6-min walking test, with 462.7 ± 6.2 m on the baseline and 492.0 ± 6.4 m after 4 months (*p* < 0.001). The results from the timed functional tests were as follows: rising from the floor test, 4.5 ± 0.3 s on the baseline and 3.8 ± 0.2 s after 4 months (*p* < 0.001); 10 meter distance running test, 4.9 ± 0.1 s on the baseline and 4.3 ± 0.1 s after 4 months (*p* < 0.001); 4-stair climbing test, 3.7 ± 0.2 s on the baseline and 3.2 ± 0.2 s after 4 months (*p* < 0.001); and 4-stair descent test, 3.9 ± 0.1 s on the baseline and 3.2 ± 0.1 s after 4 months (*p* < 0.001). Skeletal muscle quantitative MRI was performed in the pelvis and the thighs in order to assess the impact of the procedures on the muscle structure. Muscle water T2, a biomarker of disease activity, did not show any change during the training period, suggesting the absence of deleterious effects and negative impact on disease activity. Thus, a set of dynamic aerobic exercises in water can be regarded as effective and safe for patients with DMD.

## 1. Introduction

Duchenne muscular dystrophy (DMD) is one of the most severe and common forms of muscular dystrophies in childhood. It’s X-linked recessive disease and affects only boys, the incidence ranges from 3.3 to 5.5 per 10,000 newborn boys. DMD is characterized by a steadily progressing course, severe weakness, and atrophy with predominant involvement of pelvic girdle skeletal muscles, thighs, and shins, leading to early disability and loss of ability of independent movement at the age of 10–12 years (1, 2).

According to WHO recommendations, children should maintain physical activity of moderate or high intensity for not less than 60 min every day. According to the research data, boys with Duchenne muscular dystrophy do not achieve this criterion, and there is an observed activity decrease in the process of their growing up that aggravates the disease course (3, 4). In the case of the natural course of the disease, patients with DMD present negative dynamics in a 6-min distance walking test, with the results varying from −10.9 ± 69.2 meters to −25.8 ± 74.3 meters (4–6). Patients with DMD remain stable on the “plateau” phase at the age of 5–6.9 years but at the age of 8.8 ± 2.0 years, patients with DMD are characterized by negative dynamics, with test indicators varying from −0.07 to −0.33 s during a 12-month observation for rising from the floor, 10-meter running, and stairs climbing. (7–9). The control group of patients of pediatric age performs a 10-meter running test for 3.0 s on average, and a rising from the floor test and a 4-stair climbing test for 1–2 s.

Physical therapy exercises are one of the basic measures to prevent disease progression and associated orthopedic disorders (10, 11). According to international recommendations on the treatment management of patients with neuromuscular diseases, a submaximal level of movement activity is recommended. Patients are recommended to avoid eccentric strength training, as this can damage muscle fibers and accelerate the progression of the disease. This is due to a deficiency of the protein dystrophin in skeletal muscle, which has a protective effect on muscle fibers (1, 12). However, there are limited data and the majority of the studies about the effectiveness of various training programs were performed in adult patients.

One important branch of physical therapy is hydrokinesitherapy. It is a component of hydro rehabilitation that provides the dosed interchange of pressure and relaxation of the muscular system of the patient in water (in the conditions of antigravitation and hydro weightlessness) with health-improving. Therapeutic swimming is described as an excellent opportunity for physical activity for people with disabilities, improving both physical and psycho-emotional well-being. Numerous studies proved that the use of a water environment significantly relieves doing gymnastic exercises and facilitates making manipulative procedures (massage, stretching, torsion, etc.), especially in children of early age, pregnant women, weakened patients, and people with limited abilities (13).

However, there are currently no recommendations making it possible to identify an optimal exercise protocol that could be effective and safe for patients with DMD and other neuromuscular diseases (14). It is possible to accelerate the disease progression if the incorrect mode of movement activity is chosen, as there would be the possibility of injury of muscular fibers accompanied by the increase of severity of local non-specific inflammation and fibrous-fatty replacement (15, 16).

## 2. Aim

The present study aims to assess the effectiveness and safety of regular pool dynamic aerobic exercises in patients with Duchenne muscular dystrophy who are capable of independent movement.

## 3. Materials and methods

### 3.1. Patients

The study was performed at the Saint Petersburg State Pediatric Medical University where 28 patients with genetically confirmed Duchenne muscular dystrophy, who were aged 6.9 ± 0.2 years, were examined. All patient was on daily steroid treatment according DMD Care Considerations.

The study inclusion criteria were as follows:

1. Male gender.
2. Genetically confirmed diagnosis of DMD.
3. Capability of independent movement (early and late outpatient stage).
4. The last participation in rehabilitation programs with physical therapy exercises was more than 6 months before the inclusion in the study.
5. Possibility of parents/guardians and patients to visit the clinic regularly to take rehabilitation courses during the whole study period.

### 3.2. Assessment of motor functions and clinical condition

A 6-min distance walking test and timed tests, namely, rising from the floor, 10-meter running, and stair climbing and descending, were assessed on the baseline and during dynamic observation at 2 and 4 months. The assessment of muscle strength of the upper and lower extremities (bending and extension) was performed by a hand dynamometer from the dominant side (strength was measured in newtons). The assessment of the functional class of the tests’ performance was made on a six-point scale. The assessment was made in an equipped exercise therapy gym on standardized and fixed equipment and was performed by a neurologist with a completed specialized training course for the assessment by these methods. The tests were organized in equally comfortable conditions for all the patients taking into account the time of the day, convenient light clothes, and footwear. If necessary, 10–15-min breaks were allowed between the tests to avoid overfatigue. In case of physical or emotional overfatigue manifestations in a patient, the test was postponed to the next day. On the baseline and in dynamics, all patients were examined and interviewed by a neurologist to assess the most frequently described and other adverse events occurring during exercise therapy in the case of myopathy. The intensity of pain syndrome was assessed by the numeral and visual analogue scale (VAS).

### 3.3. Physical therapy regimen

The duration of the rehabilitation course was 4 months and the course was divided into two stages: preparatory and training. The training was performed under the control of an exercise therapy doctor specializing in hydro rehabilitation. The training intensity was chosen individually for each patient depending on his/her age, heart rate was calculated according to the formula of individual functional heart reserve (IFHR) = 190 - (age in years). The intensity of the preparatory stage was calculated in the following way: 51–60% of IFHR with the number of repetitions 6–8 times for each exercise. The intensity of the training stage was calculated in the following way: 61–70% of IFHR with the number of repetitions 10–12 times for each exercise. The duration of training was 60 min with a frequency of 3 times a week. All patients performed the set of exercises including: pool dynamic aerobic exercises, exercises for trunk movements in three directions, horizontal diving on the back, balance exercises using the hand-rail on the pool wall, exercises for active and passive stretching of the muscles of the shoulder girdle, back, and lower extremities in case of horizontal diving on the back and respiratory gymnastics with diving in two positions. Patients were used sports equipment - aqua-dumbbell, aqua-stick during training.

The set of exercises included:

1. Dynamic breathing exercises in water with arms spread out and with periodic full immersion in a vertical initial position. Dynamic breathing exercises during horizontal immersion in water, with the patient in the initial position lying on their back on two aqua sticks. One of the aqua sticks was placed under the shoulder blades with arms bent at the elbow joints and the other was placed under the knee joints. The patient was asked to inhale while straightening their body and exhale while bending the body at the lumbar and bringing the knee joints to the stomach.
2. Exercises with the movement of the body in three planes, with the body horizontally immersed in water and the patient lying on their back while a specialist held the patient’s head in a stable position with one hand or used two aqua sticks located in the neck and under the knees. The exercises were a series of back movements in the direction up-down and left–right, as well as rotational movements of the body around its axis. These movements were performed independently by the patient or with the help of the specialist whose hands or fingers moved along the entire spine or the paravertebral space.
3. Exercises to stretch the pectoral muscles with the arms abducted behind the back were carried out using the shoulder blades and elbows from behind, with the position fixed by the specialist’s hand or aqua-stick, with the patient horizontally immersed in water lying on their back.
4. Exercises for stretching the muscles of the lower extremities were carried out by fixing one aqua stick in the cervical spine. The patient’s arms were bent at the elbow joints, the shoulder blades were apart, and hands held the stick under water at neck level, with a second aqua stick fixed under the knees when the patient was horizontally immersed in water, lying on their back. In addition, the techniques of the breaststroke style with legs moving forward and backward were used.
5. Passive exercises for stretching the muscles of the back with the patient horizontally immersed in water, lying on their back, were carried out by fixing the occipital region of the head and the girth of the neck-collar zone of the spine with one hand, while the other hand focused on certain areas of the patient’s spine.
6. Active exercises to balance and stretch the muscles of the body using a handrail on the wall of the pool at the level of the water surface, with the patient horizontally immersed in water, lying on his back. The patient held the handrail with his hands, bent the legs at the knees and hip joints, fixed the feet so that the heels rested against the wall of the pool, and held the handrail with the toes with feet positioned shoulder-width apart. The patient, then, slowly moved their head and lays on the water while stretching their arms and placing their chest on the surface of the water.
7. Dynamic exercises where the patient side stepped along the wall to the left and right, spreading and bringing the arms to the body (breaststroke style on the back without extending the arms), alternating raising and lowering the shoulders with the arms moving above the surface of the water (crawl style on the back), and holding outstretched closed arms behind the head (arrow style on the back).

The exercises were done in a pool 130 cm deep with a water temperature of +32^o^C, рН 7.2, disinfected by active oxygen and ultra-violet radiation.

### 3.4. MR imaging and image analysis

**Figure 1:**
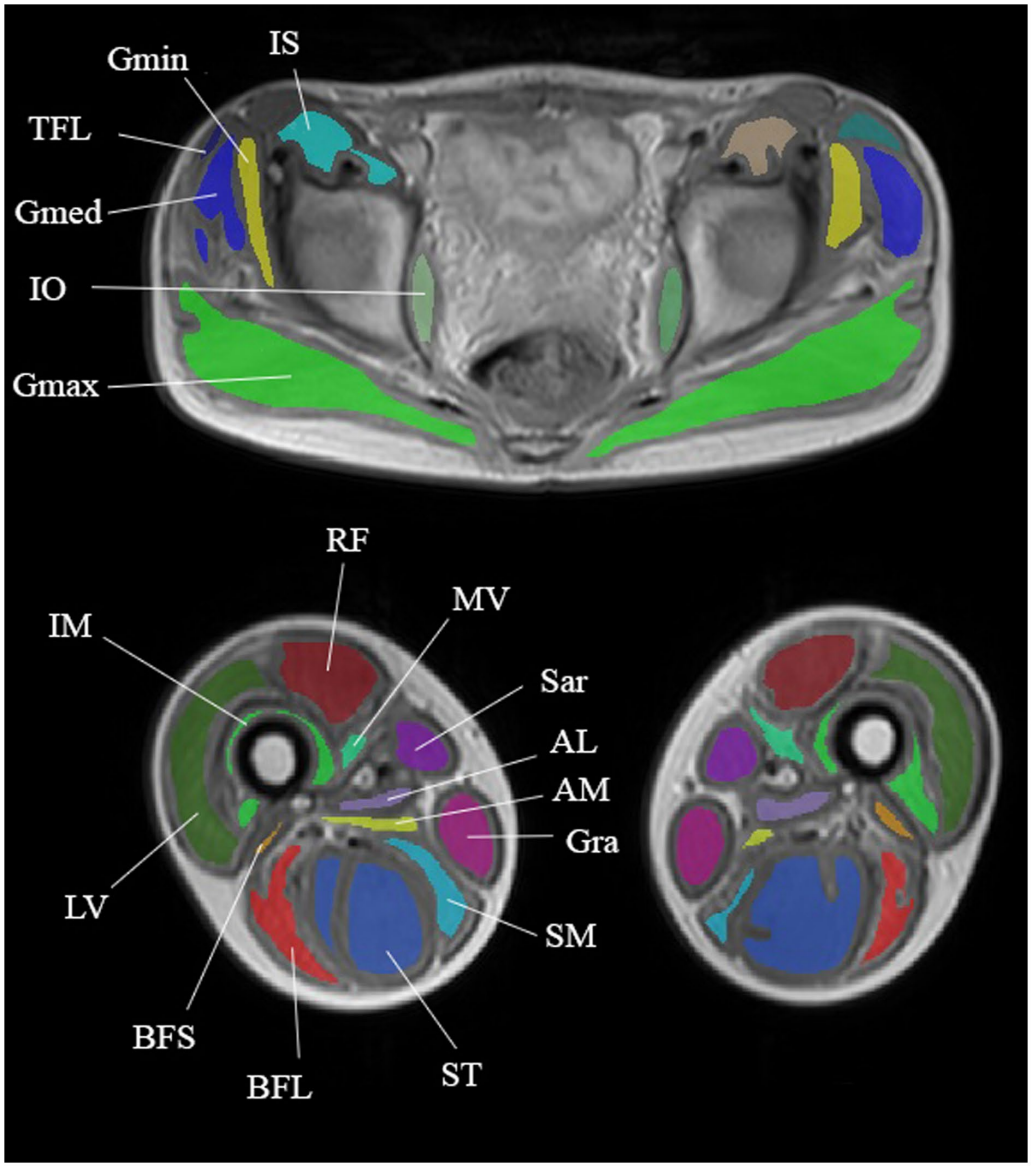
Anatomical images taken from a multi-TE SE series showing segmentation of the pelvic girdle and thigh muscles. Muscles of the pelvic girdle: gluteus maximus (GMax), gluteus medius (GMed), tensor fascia latae (TFL), iliosacral muscle (IS), and internal obturative (IO). Thigh muscles: adductor magnus (AM), quadriceps muscle (rectus femoris (RF) and lateral (LV), medial (MV), and intermediate (IM) vastus muscles), biceps femoris short (BFS) and long (BFL) heads, semitendinosus (ST), semimembranosus (SM), sartorius (Sar), and gracilis (Gra).

MRI of the pelvic girdle and thigh skeletal muscles was performed on a Philips Ingenia 1.5 T scanner; equipped with an array of receiver surface coils. A multi-TE spin echo sequence (MSME) was acquired with the following main parameters: echo train of 20 with echo times (TE) ranging between 10 and-200 ms, repetition time (TR) = 3,500 ms, excitation pulse flip angle = 90^o^, refocusing pulse angle 180^o^, number of slices = 7, distance between slices = 15 mm, slice thickness = 10 mm. Both sides were examined and measurements were made in the pelvic girdle (gluteus maximus, gluteus medius, gluteus minimus, tensor muscle of fascia lata, iliosacral muscle, external and internal obturators, and pectineus), thighs (adductor magnus, longus, and brevis, quadriceps muscle (rectus femoris, and lateral, medial, and intermediate vastus muscles), biceps femoris (short and long heads), semitendinosus and semimembranosus muscles gracilis and sartorius) (Figure 1). Six patients underwent the MRI protocol. The МRI was performed six times: before training (further BT) and after training (further AT) at baseline, after 2 and 4 months. For the MRI before training, all subjects were instructed to avoid any unusual physical activity at least 3 days before the МRI investigation. The post-training МRI was performed 3–3.5 h after the training ended. The MSME images were processed offline and the water and fat signal decays were separated using the three-exponential fit method (17), and water T2 and fat fraction maps were generated.

### 3.5. Statistical analysis

The statistical analysis was performed by software IBM SPSS Statistics v.26.0 and Microsoft Excel 2019. We performed the calculation of mean values, standard deviation, and confidence interval with α = 0.05, and paired T-Student test was performed for dependent pools. A comparison of dynamic indicators of motor abilities was carried out with the baseline level. The Pearson correlation analysis was done for the age and motor abilities of the subjects. A comparison of quantitative MRI data was carried out among the results before and after training for each of the stages.

## 4. Results

### 4.1. Dynamic of the motor functions

**Figure 2:**
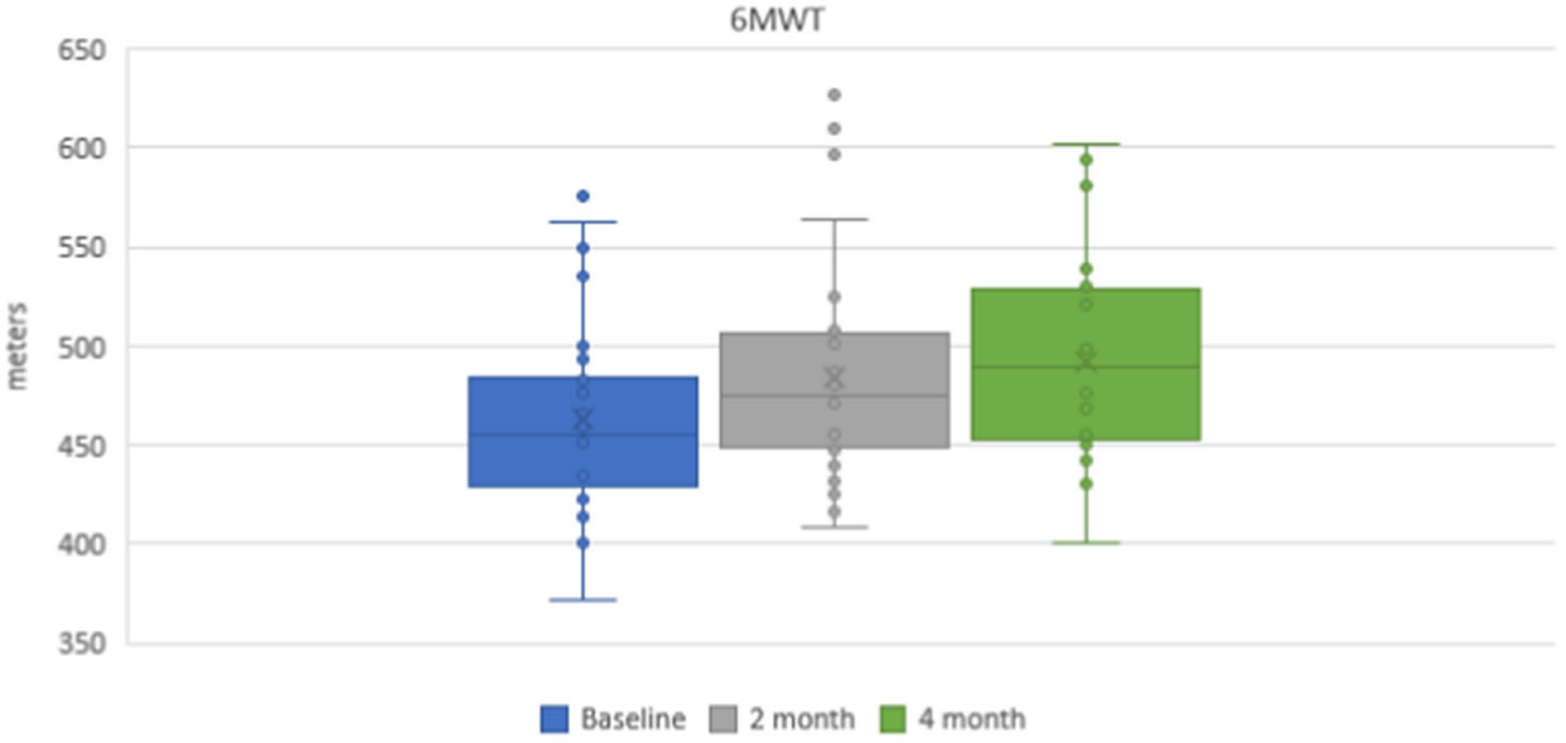
Dynamics of a 6-min walking test during 4 months.

Mean values of a 6-min walking distance were 462.7 ± 6.2 m in the group of subjects on the baseline. During repeated investigations after the course of physical therapy exercises, a statistically reliable positive dynamic was noticed and the mean distance was 484.3 ± 7.2 m (*p* < 0.001) after 2 months and 492.0 ± 6.4 m (p < 0.001) after 4 months (Figure 2).

**Figure 3:**
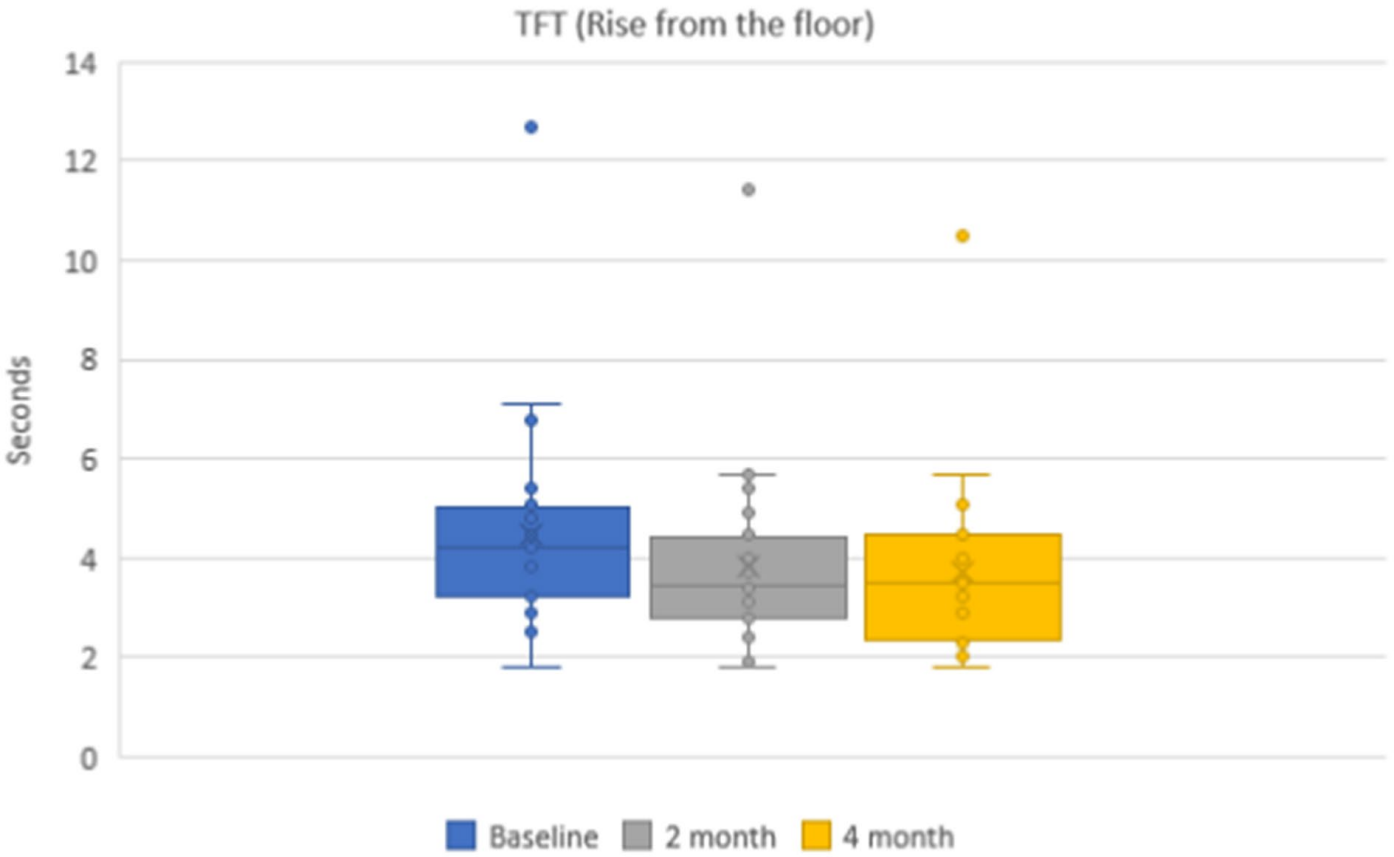
Dynamics of the timed functional test (rising from the floor) during 4 months.

Mean values of the time needed to rise from the floor were 4.5 ± 0.3 s in the group of patients on the baseline. During dynamic observation, positive dynamics were noticed, and the mean time was 3.8 ± 0.3 s (*p* < 0.001) after 2 months and 3.8 ± 0.2 s (*p* < 0.001) after 4 months (Figure 3). Mean values of functional class of rising from the floor were 3.7 ± 0.2 points on the baseline, 4.6 ± 0.1 points after 2 months, and 4.6 ± 0.1 points after 4 months.

**Figure 4:**
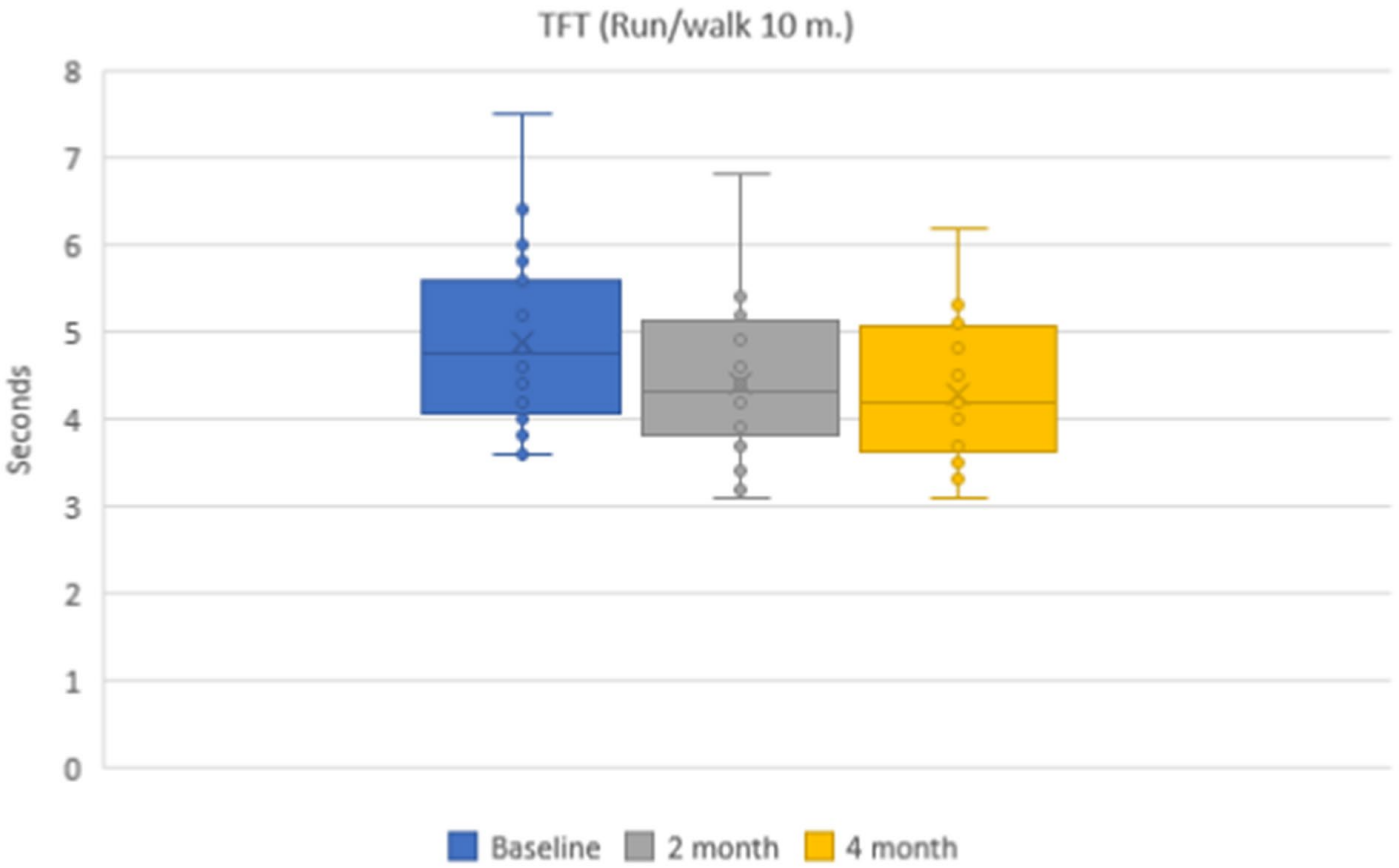
Dynamics of the timed functional test (10-meter running/walking) during 4 months.

Mean values of time needed to run 10-meter distance during a 10-meter distance running were 4.9 ± 0.1 s in the group of patients on the baseline. During dynamic observation the mean time was 4.4 ± 0.1 s (*p* < 0.001) after 2 months and 4.3 ± 0.1 s (*p* < 0.001) after 4 months (Figure 4). Mean values of functional class of rising from the floor were 5.2 ± 0.1 points on the baseline, 5.3 ± 0.1 points after 2 months, and 5.5 ± 0.1 points after 4 months.

**Figure 5:**
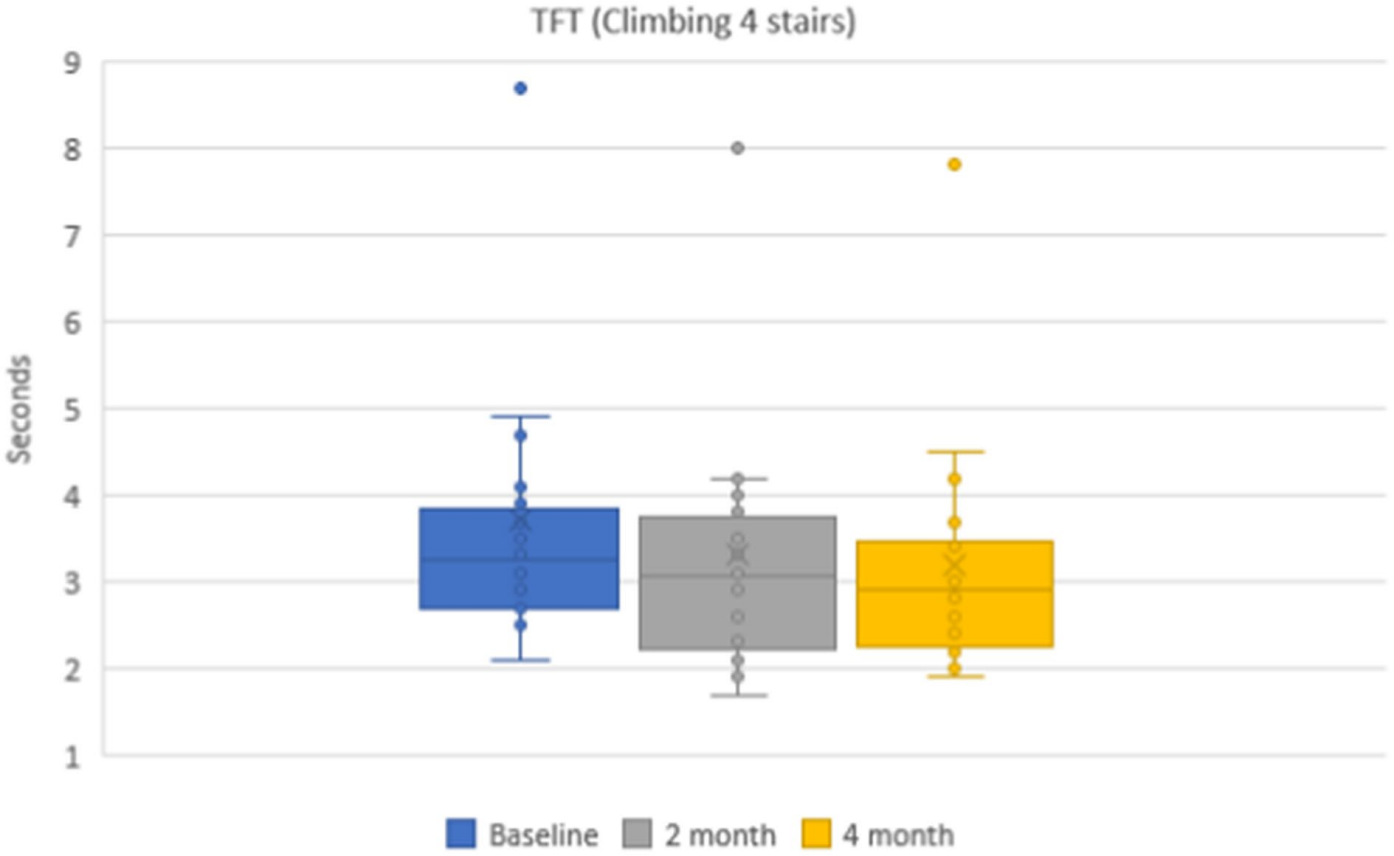
Dynamics of the timed functional test (4-stair climbing) during 4 months.

Mean values of time needed to climb 4-stairs were 3.7 ± 0.2 s on the baseline, 3.3 ± 0.2 (*p* < 0.001) after 2 months, and 3.2 ± 0.2 (*p* < 0.001) after 4 months (Figure 5). The mean values of the functional class of 4-stair climbing were without dynamics: 4.9 ± 0.2 points on the baseline and during the whole dynamic observation period.

**Figure 6:**
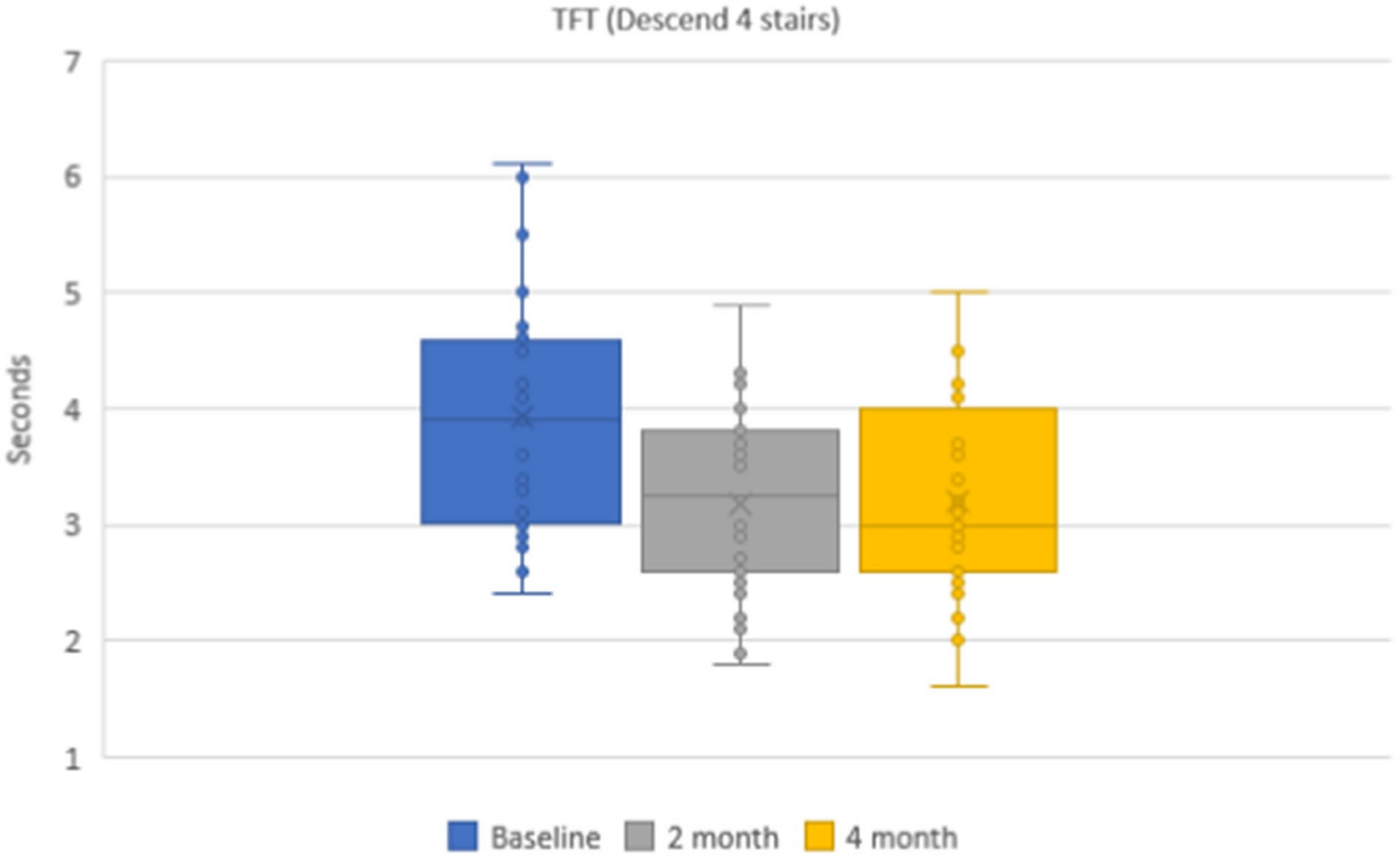
Dynamics of the timed functional test (4-stair descent) during 4 months.

The mean values of the time needed to descent 4-stairs were 3.9 ± 0.1 s in the group of patients. During dynamic observation the mean time was 3.2 ± 0.1 s (*p* < 0.001) after 2 months and 3.2 ± 0.1 s (*p* < 0.001) after 4 months (Figure 6). The mean values of the functional class of 4-stair descent were 4.4 ± 0.2 points on the baseline and 4.8 ± 0.2 points after 2 and 4 months.

Mean values of muscular strength at flexion of the leg were 47.1 ± 1.4 on the baseline, 50.9 ± 1.4 (*p* < 0.001) after 2 months, and 54.3 ± 1.6 (*p* < 0.001) after 4 months. Muscular strength at the extension of the leg was 66.8 ± 3.7 on the baseline, 77.8 ± 3.8 (*p* < 0.001) after 2 months, and 80.4 ± 4.0 (*p* < 0.001) after 4 months. Muscular strength at flexion of the arm was 41.2 ± 1.1 on the baseline, 44.5 ± 1.2 (*p* < 0.001) after 2 months, and 47.5 ± 1.2 (*p* < 0.001) after 4 months. The strength of the extension of the arm was 32.5 ± 1.3 on the baseline, whereas during dynamics it was 37.5 ± 1.6 after 2 months and 37.8 ± 1.4 (*p* < 0.001) after 4 months (Table 1). There was no correlation between the age of the subjects and distance of the 6-minute walk test (*r*=−0.117), time needed to rise from the floor (*r*=−0.131), run 10 meters (*r*=−0.180), rise and descend 4 steps (*r*=−0.175) and muscle strength (*r*=−0.124).

**Table 1:** Results of the assessment of muscular strength (newtons, N) by hand dynamometer.

	Baseline level	2 months	4 months
Flexion of the leg	47.1 ± 1.4^*^	50.9 ± 1.4^*^	54.3 ± 1.6^*^
Extension of the leg	66.8 ± 3.7^*^	77.8 ± 3.8^*^	80.4 ± 4.0^*^
Flexion of the arm	41.2 ± 1.1^*^	44.5 ± 1.2^*^	47.5 ± 1.2^*^
Extension of the arm	32.5 ± 1.3^*^	37.5 ± 1.6^*^	37.8 ± 1.4^*^

^*^p < 0,001.

### 4.2. Adverse events

During a 4-month course of physical therapy exercises, 18 patients presented adverse effects, namely, 12 (42.8%) patients had muscular pains of mild intensity and 13 (46.4%) had feelings of hardening and muscle tension in the lower extremities within 24 h after training (Table 2).

**Table 2:** Adverse events registered during the course of physical therapy within 24 h after training.

Adverse events	Frequency *n* = (%)
Muscular pain, mild (1–3 points according to VAS)	12 (42.8%)
Muscular pain, moderate (4–6 points according to VAS)	0
Muscular pain, severe (7–10 points according to VAS)	0
A feeling of consolidation and muscle tension in the lower extremities	13 (46.4%)
Increase in muscular weakness	0
Dyspnea	0
Arterial pressure disorders	0
Arrhythmia	0
Myoglobinuria	0
Traumas of joints	0
Traumas of muscles	0
Back pain	0

### 4.3. Quantitative MRI of skeletal muscles

**Figure 7:**
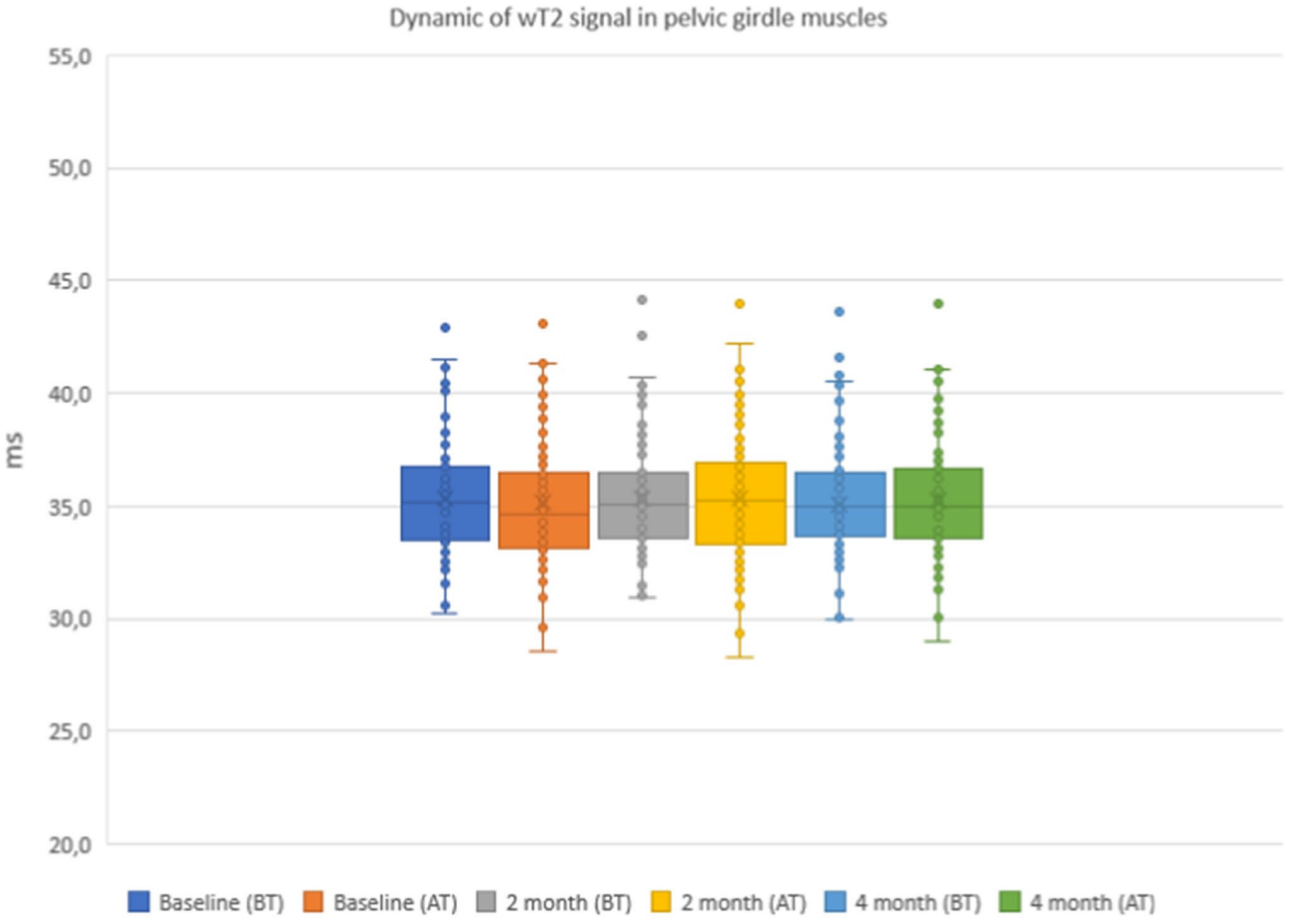
Water T2 of the patient individual pelvic girdle muscles during the 4 months of physical therapy, measured before (BT) and after (AT) training sessions.

**Figure 8:**
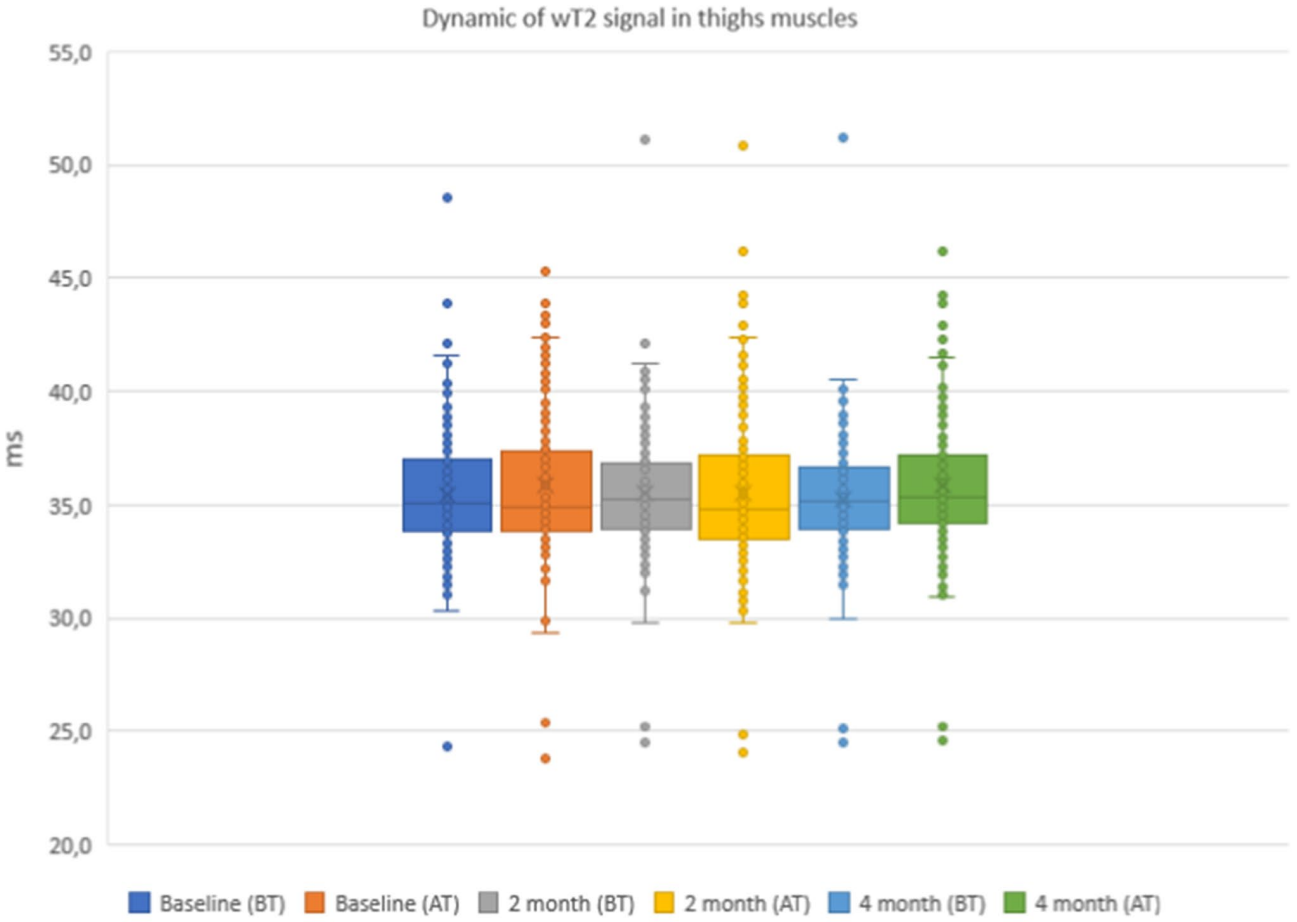
Water T2 of the patient individual thigh muscles during the 4 months of physical therapy and observation, measured before (BT) and after (AT) training sessions.

**Figure 9:**
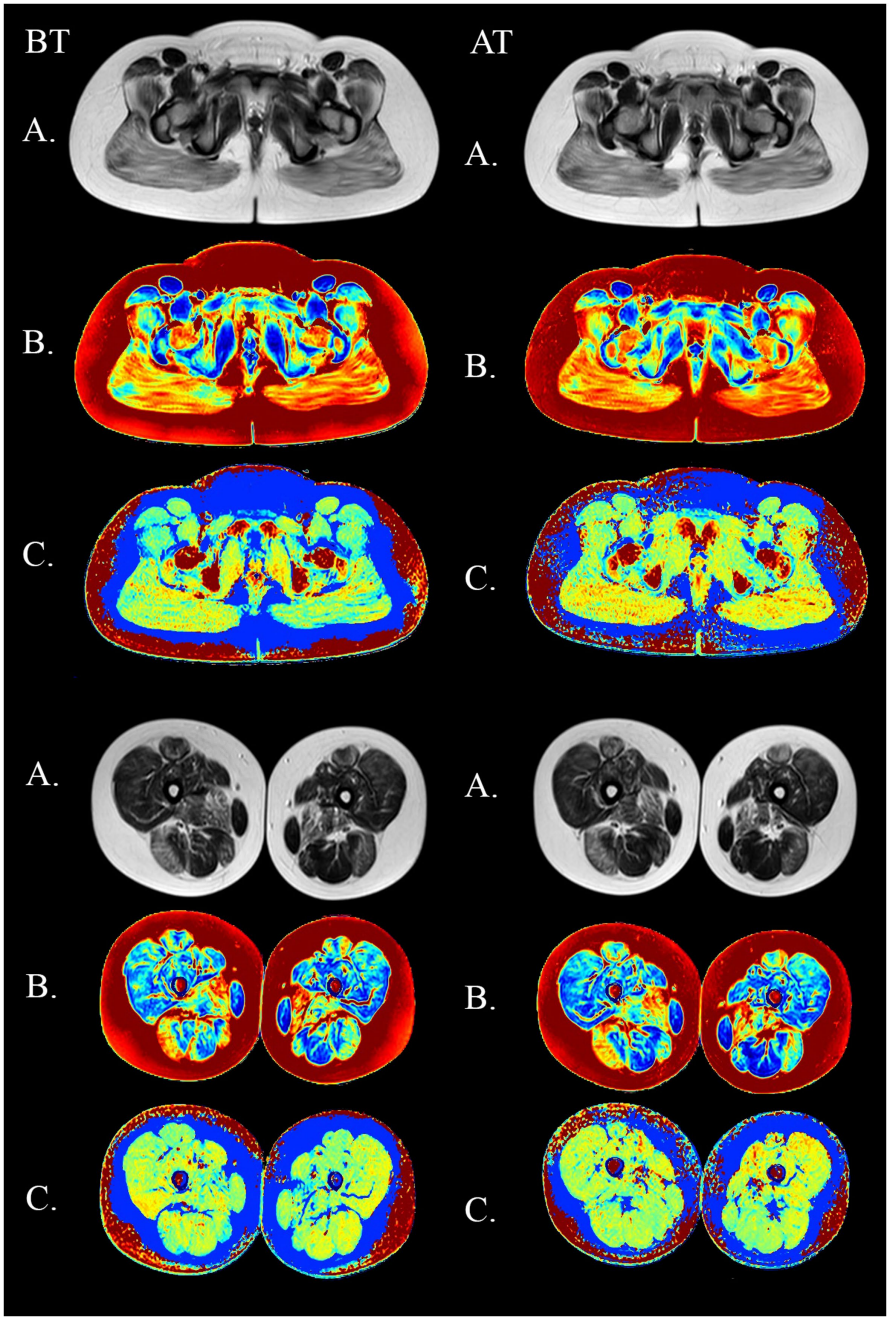
MSME imagery in a 11-year old DMD boy before (BT) and after training (AT) on the submaximal stage. Upper panel: pelvic girdle. Lower panel: thighs. **(A)** native SE images (TE = 80 ms). **(B)** fat fraction maps **(C)** water T2 maps.

The results of the quantitative MRI analyses showed that water Т2 remained constant during the course of the study, including before and after training. The mean water T2 in the pelvic girdle muscles was 35.3 ± 0.8 ms before training and 35.3 ± 0.4 ms after training at baseline, 35.2 ± 0.8 ms before training and 35.3 ± 0.8 ms after training at 2 months and 34.9 ± 0.8 ms before training and and 35.4 ± 0.8 ms after training at the end of the course (see Figure 7). In the thigh muscles, it was 35.4 ± 0.3 ms before training and 35.9 ± 0.4 ms after training at baseline, 35.5 ± 0.4 ms before training and 35.5 ± 0.8 ms after training at 2 months and 35.9 ± 0.4 ms before training and 35.9 ± 0.4 ms after training at the end of the training course (see Figure 8). More specifically, in the anterior compartment of the thighs, water T2 was measured at 35.8 ± 0.5 ms before training and 36.5 ± 0.6 ms after training at baseline, 35.9 ± 0.4 ms before training and 36.0 ± 0.6 ms after training at 2 months and 35.6 ± 0.4 ms before training and 36.3 ± 0.6 ms after training at the end of the course. In the posterior compartment of the thighs, muscle water T2 was 36.3 ± 0.6 ms before training and 36.6 ± 0.6 ms after training at baseline, 36.1 ± 0.6 ms before training and 36.1 ± 0.7 ms after training at 2 months and 35.8 ± 1.0 ms before training and 36.6 ± 0.6 ms after training at the end of the course (see Figures 8, 9). No statistically significant change was detected between before and after training water T2 measurements.

In the anterior compartment of the thigh muscles, the mean intensity of signal strength was 35.8 ± 0.5 ms before training and 36.5 ± 0.6 ms after training on the baseline, 35.9 ± 0.4 ms before training and 36.0 ± 0.6 ms after training at 2 months, and 35.6 ± 0.4 ms before training and 36.3 ± 0.6 ms after training at the end of the course.

In the posterior compartment of the thigh muscles, the mean intensity of signal strength was 36.3 ± 0.6 ms before training and 36.6 ± 0.6 ms after training at baseline, 36.1 ± 0.6 ms before training and 36.1 ± 0.7 ms after training after 2 months, and 35.8 ± 1.0 ms before training and 36.6 ± 0.6 ms after training at the end of the course. There were no statistically significant changes when comparing before and after training. The details are given in Figures 7–9.

## 5. Discussion

In our study, we evaluated the motor abilities of patients with DMD regularly doing pool aerobic exercises for 4 months. In the natural course of the disease, the physical abilities of patients are characterized by a “plateau” phase at early stages and by negative dynamics at later stages (1). After regular courses of physical exercises were introduced for those patients who had not previously had a physical load for a long time, we noticed positive dynamics with improvement in the 6-min distance walking test, dynamometry, and timed tests. These are evidence of improvement of the cardiorespiratory system due to regular dynamic aerobic and respiratory exercises, as well as to the improvement of indicators of muscular strength. There was no statistically significant correlation between the age of patients and motor abilities. This allows to draw a conclusion about the effectiveness of exercises at all ambulatory stages. The observed changes may be due to dynamic gymnastics being a movement accompanied by the interchange of muscle pressure and relaxation, providing an aerobic way of energy supply. It occurs due to the increase of sympathetic nervous system activity and significant homeostatic effect, which is based on the improvement of hemocirculation and metabolic processes (18, 19). The combination of motor reaction speed and frequency of heart rate is the base of physiological reactions in dynamic gymnastics (18). A proper response to physical activities is accompanied hemodynamical improvement. The consequence of such training is a significant increase in the functional abilities of the cardiorespiratory system (20). Key-features of dynamic gymnastics are the following: (1) Training of muscle high-speed endurance that depends mainly on the speed of movements but not on the duration of load. Speed training at an early age (under 7 years of age) promotes the increase of speed of movement reactions that have congenital character, (2) Muscular mass increase occurs due to the increase of myofibrils numbers. Muscular fibers are usually oriented parallel to the muscle long axis. There is an elongation of the muscular part and a shortening of the tendinous part of the muscular fiber. The increase in muscle capacity is greater than in the case of isometric gymnastics, and (3) dynamic load promotes stimulation and elongation of tubular bones (19–21).

At present, there is limited data about the effectiveness of aqua-aerobics in patients with neuromuscular diseases, including DMD. According to some authors, regular pool exercises promote a considerable improvement in the strength and endurance of respiratory muscles, PCF, Sat O2, and tolerance to physical activity (22, 23). According to the results of other studies, regular aerobic exercises in physical therapy gym with an instructor, in combination with exercise bicycle training, also provide improvements in motor functions in patients with DMD. An in the number of pedal turns during exercise bicycle training in 30 patients with DMD was identified after 24 weeks of aerobic training. In addition, the authors showed the improvement of arm motor functions in those patients who were not able to independently move on foot (24). An important advantage of water exercises is the big adherence of the majority of patients and parents, which has a positive effect on the child’s emotional condition, as was identified in our study and in many other observations (25).

Safety is an important aspect concerning physical exercise in cases of DMD. One of the basic pathogenetic mechanisms of DMD is inflammatory changes with increased activity of macrophages and leukocytes in skeletal muscles (15). Local non-specific inflammatory changes are triggered by the damage caused to muscle fibers as a result of myocyte membrane leakiness and fragility in the absence of dystrophin protein synthesis. This leads to multiples cycles of myocyte degeneration and regeneration, ultimately resulting in fibrous-fatty replacement (16, 26, 27), which has been demonstrated in a number of previous studies and has a high correlation with the motor abilities of patients with DMD in the ambulant stages of the disease (28–30). An increase in water T2, detected qualitatively with STIR T2 weighted sequences or measured with T2 maps, as in this study, is a biomarker of the disease activity and may be a predictor of further fibro-fatty degeneration of the muscles (31).

In our study population, muscle water Т2 was increased in all muscle groups that were investigated, as expected from the results of a previous investigation (32). The predominance of muscle damage in the quadriceps has also been described in the natural course of the disease at the early ambulatory stages (33), which probably makes these muscles the most vulnerable to additional physical stress.

We occasionally observed local and circumscribed T2 hyperintensities in some muscles of the thighs. It did not translate into statistically significant changes in muscle water dynamics before and after activity. The absence of significant edema and inflammation induced by exercise represents an additional and important biomarker of safety for the proposed physical therapy protocol, with the advantage of assessing muscle integrity locally.

During the course of training, all patients were followed by a neurologist to identify adverse effects associated with the exercises performed. All registered adverse effects (muscular pain and feeling of tension in leg muscles) are natural in the course of DMD and were mild and short lasting. No adverse effects resulted in the refusal of parents and patients to participate in the training or caused an inability to continue the course of training.

## 6. Conclusion

Regular aqua-aerobic exercises in a short-term prospect of 4 months have a complex influence on the organism, with a training influence on cardiorespiratory and muscular systems and promoting the improvement of indicators of strength and endurance in patients with DMD who are capable of independent movement. Based on skeletal muscle MRI and clinical observation, these exercises can be considered safe. Nevertheless, it's important to observe long-term effect of the exercises and speed of the disease progression.

## Data availability statement

The original contributions presented in the study are included in the article/supplementary material, further inquiries can be directed to the corresponding author.

## Ethics statement

The studies involving human participants were reviewed and approved by Ethical Committee of Saint Petersburg State Pediatric Medical University. Written informed consent to participate in this study was provided by the participants’ legal guardian/next of kin.

## Author contributions

VS, PC, GP, DR, and GS took part in the study’s conception and design. LL and EA were responsible for training and supervising the subjects during the rehabilitation course. VS and LL participated in data acquisition. VS, PC, and GP performed data analysis, interpretation, and statistical analysis. PC, VS, and DI contributed to significantly revising the manuscript. All authors contributed to the article and approved the submitted version.

## Conflict of interest

The authors declare that the research was conducted in the absence of any commercial or financial relationships that could be construed as a potential conflict of interest.

## Publisher’s note

All claims expressed in this article are solely those of the authors and do not necessarily represent those of their affiliated organizations, or those of the publisher, the editors and the reviewers. Any product that may be evaluated in this article, or claim that may be made by its manufacturer, is not guaranteed or endorsed by the publisher.
